# Dietary behaviours during pregnancy: findings from first-time mothers in southwest Sydney, Australia

**DOI:** 10.1186/1479-5868-7-13

**Published:** 2010-02-03

**Authors:** Li Ming Wen, Victoria M Flood, Judy M Simpson, Chris Rissel, Louise A Baur

**Affiliations:** 1 School of Public Health, Sydney Medical School, University of Sydney, Australia; 2 Health Promotion Service, Sydney South West Area Health Service, New South Wales, Australia; 3 Cluster for Public Health Nutrition, University of Sydney, Australia; 4 Discipline of Paediatrics & Child Health, University of Sydney, Australia

## Abstract

**Background:**

Limited prevalence data are available for nutrition related health behaviours during pregnancy. This study aimed to assess dietary behaviours during pregnancy among first-time mothers, and to investigate the relationships between these behaviours and demographic characteristics, so that appropriate dietary intervention strategies for pregnant women can be developed.

**Method:**

An analysis of cross-sectional survey was conducted using data from 409 first-time mothers at 26-36 weeks of pregnancy, who participated in the Healthy Beginnings Trial conducted in southwestern Sydney, Australia. Dietary behaviours, including consumption of vegetables, fruit, water, milk, soft drinks, processed meat products, fast foods/take away and chips, were assessed using the New South Wales Health Survey questionnaire through face-to-face interviews. Factors associated with dietary behaviours were determined by logistic regression modeling. Log-binomial regression was used to calculate adjusted risk ratios (ARR).

**Results:**

Only 7% of mothers reported meeting the recommended vegetable consumption and 13% reported meeting the recommended fruit consumption. Mean and median intakes per day were 2.3 (SD 1.3) and 2 serves of vegetables, and 2.1 (SD 1.4) and 2 serves of fruit respectively. About one fifth of mothers (21%) reported drinking 2 cups (500 ml) or more of soft drink per day and 12% reported consuming more than 2 meals or snacks from fast-food or takeaway outlets per week. A small percentage of mothers (5%) had experienced food insecurity over the past 12 months. There were significant inverse associations between water and soft drink consumption (Spearman's ρ -0.20, P < 0.001), and between fruit and fast food/takeaway consumption (Spearman's ρ -0.16, P = 0.001). The dietary behaviours were associated with a variety of socio-demographic characteristics, but no single factor was associated with all the dietary behaviours.

**Conclusions:**

There were low reported levels of vegetable and fruit consumption and high reported levels of soft drink and takeaway/fast food consumption among pregnant women. Dietary interventions to prevent adverse health consequences need to be tailored to meet the needs of pregnant women of low socio-economic status in order to improve their own healthy eating behaviors. Increasing water and fruit consumption could lead to reduced consumption of soft drink and takeaway/fast food among pregnant women.

**Trial Registration:**

HBT is registered with the Australian Clinical Trial Registry (ACTRNO12607000168459)

## Introduction

Nutrition during pregnancy is a significant public health concern. Poor nutrition can lead to a range of health problems for mothers, including cardiovascular disease, diabetes, cancer, and overweight and obesity [1-5]. Lack of adequate nutrition of pregnant women to the growing fetus is a key causal factor for stillbirths prior to the onset of labour [6]. Pregnancy is characterised by additional energy requirements of approximately 300 kcals (or 1256 KJ) per day [7], with energy metabolism changing during pregnancy and varying considerably among women [8]. Thus, healthy nutrition intake becomes critical for the health of the mothers and their infants. The current Australian national dietary guideline recommends four serves of fruit and five to six serves of vegetables daily during pregnancy [9,10].

Poor nutrition that results from an inadequate dietary intake is associated with a range of social, economic and cultural factors [11-14]. Some measures of poor nutrition include eating less fruit and vegetables than recommended. In the state of New South Wales, Australia, only around ten per cent of the general population report having consumed the recommended five or more serves of vegetables a day [15]. Just over half of the population report having consumed the recommended two or more serves of fruit [15], but the true figure could be markedly lower as a validation study suggests that the perceived size of a serve of vegetables as reported by the general population is much less than the actual size of a recommended serve [16,17]. It is evident that social-economic status (SES) plays an important role in nutritional intake by individuals. In general, nutritional intake among a population with higher SES tends to be closer to the dietary recommendations [12,14].

To date, however, limited prevalence data are available for nutrition related health behaviours during pregnancy and most research of this kind is focused on specific nutrients related to dietary concerns [18-22]. This has created a challenge for health promotion or healthcare providers in developing dietary behaviour interventions appropriate to the needs of pregnant women. The only recent Australian study found that few pregnant women met the guidelines for recommended fruit (9%) and vegetables (3%) intake [23]. That study recommended that research investigating patterns of occurrence of multiple risk factors for unhealthy behaviours, particularly stratification by SES would provide more information for planning interventions [23]. Therefore, a better understanding of dietary patterns during pregnancy, which could facilitate the development of practical public health interventions, is urgently needed.

In 2008, we commenced the Healthy Beginnings Trial (HBT) to test the effectiveness of an early childhood obesity intervention in the first two years of life [24]. The intervention uses a home-visiting strategy to promote healthy feeding of babies among first-time mothers. One of the objectives of the trial is to increase mothers' healthy eating for themselves and healthy feeding for their infants. In this particular study, we aim to assess dietary behaviours during pregnancy of first-time mothers, and to investigate the associations between these behaviours and demographic characteristics, so that appropriate nutritional intervention strategies can be developed.

## Methods

### Study design

A cross-sectional survey was conducted in 2008 as part of the baseline data collection of the HBT. The study was conducted in southwestern Sydney, Australia and approved by the Ethics Committee of Sydney South West Area Health Service. The details of the HBT research protocol have been reported elsewhere [24].

### Study participants

A total of 667 first-time mothers at 26-36 weeks of pregnancy, who attended antenatal clinics at Liverpool or Campbelltown Hospitals, located in south-western Sydney, were recruited for the HBT. They were approached by research nurses with a letter of invitation and information about the study. First-time mothers who were able to communicate in English were eligible for the trial. Once eligibility was established and consent obtained, mothers were asked to fill in a registration form with their contact information to allow the nurses to make further arrangements for the baseline data collection. Four hundred and nine mothers were interviewed at their home before giving birth and were included in this particular study. Another 258 mothers who also participated in the HBT were excluded, as we were not able to conduct the survey before they gave birth.

### Data collection and key measures

A face-to-face interview with each participating mother was conducted by one of four research nurses. The interview was of 20 to 30 minutes' duration and included a range of questions about general health, physical activity, dietary behaviours and food insecurity.

Mothers also reported their pre-pregnancy weight and height, and we calculated body mass index (BMI) as weight/height^2 ^(kg/m^2^). Maternal BMI was then categorised as underweight (<18.50 kg/m^2^), healthy weight (18.50 - 24.99 kg/m^2^), overweight (25.00-29.99 kg/m^2^), or obese (≥ 30 kg/m^2^).

To assess mothers' dietary behaviours during pregnancy, a set of dietary questions sourced from the NSW Health Survey Program, Australia [15] were asked including daily consumption of vegetables, fruit, water, soft drinks, and milk, as well as frequency of intake of processed meat, meals from fast food outlets or local takeaways and chips or French fries. The majority of these questions have been validated in an adult population and are widely used in NSW population health surveys [16,17]. The validity of these short questions generally shows an ability to rank-order food intake but response categories do not usually provide good estimates of absolute quantities of intake [17]. Some of the questions used in the study are listed below as examples.

How many serves of vegetables do you usually eat each day? (Include fresh, dried, frozen and tinned vegetables. One serve = 1/2 cup cooked or 1 cup of salad vegetables)

How many serves of fruit do you usually eat each day? (Include fresh, dried, frozen and tinned fruit. One serve = 1 medium piece or 2 small pieces of fruit or 1 cup of diced pieces)

How many cups of soft drink, cordial, or sports drink, such as lemonade or Gatorade, do you usually drink in a day? (One cup = 250 ml. 1 can of soft drink = 1 1/2 cups. 1 × 500 ml bottle of Gatorade = 2 cups) (Do not include diet drinks)

How often do you have meals or snacks such as burgers, pizza, chicken, or chips from places like McDonalds, Hungry Jacks, Pizza Hut, KFC, Red Rooster or local takeaway food places?

In addition, age, employment status, education level, marital status, language spoken at home, and country of birth, were obtained using standard questions from the NSW Health Survey [15].

### Analysis

Statistical analyses were carried out using Stata 10 [25]. Descriptive statistics including mean and median for various dietary intakes were calculated. Consumption frequencies were then categorised and proportions were compared between groups using Pearson chi-squared tests or Mantel-Haenszel chi-squared tests for trend in proportions when appropriate.

Spearman's rank correlation, rho (ρ), was used to examine the associations between water or milk consumption and soft drink or fruit juice consumption, and between fruit or vegetables and having takeaway/fast food or eating chips/fries.

To examine the predictors of poor nutrition behaviours, four logistic regression models were developed, one for vegetable consumption <2 serves/day, one for fruit consumption <2 serves/day, one for having >1 cup of soft drink/day and one for having takeaway/fast food >2 times/week. Variables with P < 0.25 on bivariate analysis were entered into each model, then the least significant terms were progressively dropped until only those with P < 0.05 and those that confounded the effect of these variables remained in the model. Adjusted risk ratios (ARRs) with 95% confidence intervals were calculated by refitting the final models using log-binomial regression with the Stata binreg command.

## Results

The main characteristics of the participating mothers are shown in Table 1. The age range of the mothers was from 16 to 46 with a mean of 26 years. Most of the mothers (87%) were either married or living with their de facto partner. Twenty three per cent had completed tertiary education and 11% spoke a language other than English at home. In addition, 22% were unemployed and 29% had a household income before tax of less than $40,000 per year.

**Table 1:** Characteristics of the 409 participating first-time mothers

Variables	n (%)
**Age**	
≤ 24	175 (43)
25-29	139 (34)
≥ 30	95 (23)
**Marital status**	
Married/de-facto partner	354 (87)
Never married	53 (13)
**Employment status**	
Employed/paid and unpaid maternity leave	224 (55)
Unemployed	88 (22)
Home duties/Student/other	96 (23)
**Education**	
Completed primary school to school certificate	86 (21)
HSC to TAFE certificate or diploma	229 (56)
University/tertiary	92 (23)
**Country of birth**	
Australia	274 (67)
Other	134 (33)
**Language spoken at home**	
English	364 (89)
Other	44 (11)
**Household income**	
<$40,000	121 (29)
$40,000-$79,999	138 (34)
≥ $80,000	150 (37)
**Weight status based on BMI (self-reported)**	
Underweight	28 (7)
Normal	214 (55)
Overweight	97 (25)
Obese	49 (13)
**Food insecurity**	
Yes	21 (5)
No	388 (95)
**Reported meeting recommended vegetable consumption **(≥ 5 serves/day)	
Yes	27 (7)
No	382 (93)
**Reported meeting recommended fruit consumption **(≥ 4 serves/day)	
Yes	53 (13)
No	356 (87)

* HSC = Higher School Certificate, TAFE = Technical And Further Education

Table 1 also shows that 38% of the mothers were either overweight (25%) or obese (13%) prior to pregnancy, based on reported BMI. Only 7% of the mothers reported meeting the recommended daily vegetable consumption (≥ 5 serves) and 13% reported meeting the recommended fruit consumption (≥ 4 serves). In addition, 5% of mothers reported having experienced a time in the past 12 months when they ran out of food and could not afford to buy more (a measure of food insecurity).

### Dietary behaviours

The distributions of dietary behaviours of the first-time mothers stratified by age, education and household income are shown in Additional File 1: Table S1. The mean and median daily vegetable consumption were 2.3 (SD 1.34) and 2 serves respectively. One third of mothers (33%) reported having less than 2 serves of vegetables per day, and among those with a lower household income (<$40,000) a higher proportion ate few vegetables (46%, P = 0.001). With an average daily consumption of 2 serves of fruit there were 11% of mothers having no fruit or less than 1 serve/day, in particular among younger mothers (19%, P = 0.002).

The mothers reported drinking an average of 6.1 cups (SD 3.4) (1 cup = 250 ml) or 1.5 litres of water and 1.5 cups (SD 1.6) of fruit juice daily. The mean and median of soft drink consumption were 1.5 cups (SD 2.1) and 1 cup respectively, and 38% reported consuming more than 1 cup of soft drink per day. Higher proportions of younger mothers (43%, P < 0.001) and less educated mothers (44%, P < 0.001) reported drinking more than 1 cup of soft drink per day. Average daily reported milk consumption for all the mothers was 2.1 cups (SD 1.51), but 39% had less than 1 cup a day.

One third of the mothers (33%) reported having breakfast cereal including ready-made, home-made or cooked cereal no more than 2 times per week, and 37% of the mothers had eaten pasta, rice or noodles no more than 2 times per week. Interestingly, mothers with tertiary education were less likely to have breakfast cereal more than 6 times per week (37% vs 48%, P = 0.03) but more likely to have pasta, rice or noodles more than 6 times per week (43% vs 21%, P < 0.001).

About 40% of the mothers reported having processed meat products such as sausages, frankfurts, devon, salami, meat pies or ham, more than once a week regardless of maternal age, education or household income. In contrast, younger or less educated mothers, or mothers with lower household income, reported having meals or snacks from fast-food outlets (e.g. McDonalds, KFC) or local takeaway shops more often (>2 times per week). In addition, younger or less educated mothers reported having chips, fries or wedges more often (>2 times per week) than the rest.

### Relationships between various dietary behaviours

As shown in Table 2, there were significant inverse associations between water consumption and drinking fruit juice (ρ = -0.17, P = 0.001) or soft drinks (ρ = -0.20, P < 0.001), and between drinking water and sugary drinks (including soft drink and fruit juice) (ρ = -0.26, P < 0.001). The relationships between milk consumption and other drinks were not significant.

**Table 2:** Spearman's rank correlation between dietary behaviours

	Water		Milk			
				
	ρ	P	ρ	P		
**Fruit juice**	-0.170	0.001	0.004	0.94		
**Soft drinks**	-0.203	<0.001	0.030	0.56		
**Diet soft drinks**	0.078	0.17	0.004	0.95		
**Sugary drinks**	-0.257	<0.001	0.047	0.35		
**Milk per day**	0.019	0.71				
						
	**Vegetables**		**Fruit**		**Soft drinks**	
			
	**ρ**	**P**	**ρ**	**P**	**ρ**	**P**

**Cereal per week**	-0.026	0.60	0.068	0.17	0.091	0.07
**Pasta per week**	-0.047	0.35	0.118	0.02	-0.046	0.37
**Processed meat per week**	0.023	0.65	-0.163	0.001	0.236	<0.001
**Fast food/takeaway per week**	-0.082	0.10	-0.164	0.001	0.237	<0.001
**Chips per week**	-0.031	0.54	-0.131	0.008	0.262	<0.001
**Vegetables per day**			0.131	0.008	-0.037	0.47

**ρ**: Spearman's rank correlation coefficient

Table 2 also shows that there were inverse associations between fruit consumption and frequency of having processed meat (ρ = -0.16, P = 0.001), having fast food/takeaway (ρ = -0.16, P = 0.001) or having chips (ρ = -0.13, P = 0.008). Fruit consumption was also positively correlated with pasta (noodles, rice) consumption (ρ = 0.12, P = 0.02) and vegetable consumption (ρ = 0.13, P = 0.008).

In addition, soft drink consumption was significantly correlated with the frequency of having processed meat (ρ = 0.24, P < 0.001), eating fast-food/takeaway (ρ = 0.24, P < 0.001), or having chips (ρ = 0.26, P < 0.001).

### Socio-demographic characteristics and poor dietary behaviours

Associations between socio-demographic variables and poor dietary behaviours in multivariate analysis using logistic regression and log-binomial regression modelling are shown in Table S2 (see Additional file 2: Table S2). Household income was significantly associated with vegetable consumption. Mothers with a higher household income (≥$80,000) were less likely to consume few serves of vegetables (<2 serves/day) compared with those with a lower income (ARR 0.6, 95% CI 0.4 to 0.9, P = 0.003), after adjusting for the confounding effect of mother's employment status.

Mother's education level was independently associated with daily fruit consumption after adjusting for mother's country of birth, with tertiary educated mothers being less likely to consume <2 serves of fruit than less educated mothers (ARR 0.6, 95% CI 0.4 to 0.8, P = 0.03). In addition, a higher education level was a protective factor against consuming soft drinks. Mothers with tertiary education were significantly less likely to report consuming >1 cup of soft drink per day than those with a lower level of education (ARR 0.3, 95% CI 0.2 to 0.6, P = 0.001).

Country of birth was also found to be independently associated with soft drink consumption. Mothers who were born outside Australia were more likely to report consuming >1 cup of soft drink per day (ARR 1.8, 95% CI 1.1 to 3.1, P = 0.03) after adjusting for education level.

Finally, mother's age and marital status were significantly associated with frequency of fast-food/takeaway consumption. Older mothers were less likely to have fast-food/takeaway more than 2 times per week compared with younger mothers (ARR 0.4, 95% CI 0.1 to 0.9, P = 0.03), but single mothers were more likely to have fast-food/takeaway more than 2 times per week (ARR 1.9, 95% CI 1.1 to 3.7, P = 0.04).

## Discussion

This cross-sectional study conducted with first-time mothers at 26-36 weeks of pregnancy in southwestern Sydney indicates that there are low proportions of mothers who report meeting the recommended intake of vegetables or fruit during pregnancy. More worryingly, there are significant proportions of mothers who reported consuming more than 2 cups (500 ml) of soft drink a day and have fast-food/takeaway more than 2 times a week. Significant inverse associations were found between the intake of water and soft drinks, and between fruit consumption and eating fast-food/takeaway, processed meat or chips. Soft drink consumption was also found to be positively correlated with fast-food/takeaway consumption. In addition, this study found that a range of poor dietary behaviours was associated with various demographic factors, although no single factor was associated with all the dietary behaviours.

To our knowledge, this is the first time that a range of dietary behaviours, rather than nutrient intake [18-21], of first-time mothers in mid to late pregnancy, have been investigated in Australia. Our findings support the conclusion of a previous study, from Queensland, that few pregnant women report meeting the guidelines for recommended fruit and vegetables intake [23], even though another study from Japan suggests that pregnant women are aware of the need to adopt healthy behaviours [21].

The positive correlation between soft drink consumption and eating fast/takeaway food in this study is consistent with other studies linking soft drink intake and poor dietary quality, such as a higher intake of saturated fat and a lower intake of key nutrients [26,27]. However, in contrast to a previous study suggesting that soft drink consumption was negatively related to milk intake among mothers [28], we found no association between milk intake and soft drink consumption. This difference may be explained by the use of different dietary measures in the two studies, as the measurement of milk intake in our study covered a broad milk consumption, including milk used in tea, or coffee, cow milk, soy milk, milk on cereal and flavoured milks and was based on a set of short questions assessing dietary behaviours [15].

Without doubt soft drink consumption is one of the key modifiable dietary behaviours that need to be targeted in the prevention of poor nutrition and obesity [29]. The inverse relationship between water and soft drink consumption, and between fruit and other takeaway/fast food suggests that encouraging water and fruit consumption could potentially lead to reduced consumption of soft drink and other takeaway/fast foods.

Our findings are consistent with other research in the general population linking dietary behaviours to SES and other demographic factors [11-14]. While most studies suggest SES and education are the stronger predictors of nutrient intake [11,12,14,30], we found that some socio-demographic characteristics were associated with particular dietary behaviour. For example, household income was associated with vegetable consumption, mother's education level was associated with daily fruit consumption and mother's country of birth was associated with soft drink consumption. In addition, mother's age and marital status were associated with fast-food/take away consumption. However, there was no single demographic factor associated with all the dietary behaviours we examined. This is an important consideration when developing dietary interventions, as tailored interventions to meet the needs of specific demographic sub-groups of pregnant women may be more successful. Specific demographic characteristics of the target population should be considered in the development of a dietary intervention.

In addition, the study found that, even using self-reported weight status, which is likely to give an underestimate [31], there was a high prevalence of overweight and obesity in women prior to their pregnancy - 38% of mothers were either overweight (25%) or obese (13%). The overall rate of combined overweight and obesity is similar to the self-reported rate of women in the same age range in NSW, but the rate of obesity is slightly higher than that of NSW women [15]. Although weight status and its relationships with dietary behaviours and socio-demographic factors are not a focus of this study, it is understood that maternal weight status and dietary behaviours play an important role in the future health of infants. These in turn are also likely to be influenced by mothers' socio-demographic factors. With an increased need for early dietary interventions that establish healthy feeding practices and patterns of healthy eating among pregnant women in order to prevent early onset of childhood overweight and obesity [5,24], a good understanding of these relationships is warranted.

### Limitations of this study

There are a number of limitations of the study. First, the selection of study participants may limit the generalisability of the study findings, given that the participating mothers were able to communicate in English. Second, the locality of the study area, southwestern Sydney, is one of the most socially and economically disadvantaged area of metropolitan Sydney, Australia [15], which may give rise to a range of potential confounders for which we were unable to control. Third, we may not have included other specific dietary behaviour changes that are important for nutrition in pregnancy. Fourth, due to the cross-sectional nature of this study, no casual relationships should be asserted between the variables we examined. In addition, there are differences in dietary assessment tools between different studies, as a result of which it is difficult to make any comparisons across studies. The inconsistencies found in different studies suggest that more research, particularly with standardised measurement tools for assessing dietary behavours, should be conducted to ascertain the levels of dietary intake and relationships between various dietary behavours, and between dietary behaviours and socio-demographic factors.

Caution also needs to be exercised when interpreting these self-reported data on dietary behaviours. In terms of recommended serves, the questions we used in this study do give reasonable rank-order validity but the quantities reported tend to be different in terms of absolute amounts as suggested by the validation studies [16,17]. We cannot rule out the possibility that the mothers in this study over-estimated the perceived size of a serve of vegetable or fruit intake. As a result, the findings from this study that 7% of mothers reported meeting the recommended vegetable consumption and 13% reported meeting the recommended fruit consumption could be over-estimated

Traditionally, dietary assessment methods have been known to under-report energy intake [32] and therefore under-report the foods which are more energy dense. It has also been observed that food frequency questionnaires (FFQs) have a tendency to over-report fruit and vegetable consumption (less energy dense foods). This has been observed among both long FFQs [33,34] and short FFQs [16,17]. It is therefore more likely that the mothers may have over-estimated the perceived size and amount consumed of a vegetable or fruit, rather than under-estimated, but we do not have specific data on this.

## Conclusions

There were low levels of vegetable and fruit consumption and high levels of soft drink and takeaway/fast food consumption among pregnant women. The dietary behaviours were influenced by various socio-demographic characteristics. Recognising the health issues associated with dietary behaviours and their associations with socio-demographic characteristics, as provided by this study, is important for the development of dietary interventions to prevent adverse health consequences among pregnant women. Increasing water and fruit consumption could potentially lead to reduced consumption of soft drink and takeaway/fast food among pregnant women.

## Competing interests

The authors declare that they have no competing interests.

## Authors' contributions

In this study, LMW undertook literature review, data analysis and interpretation and wrote the original draft. JS provided advice on data analysis. VF, JS, CR and LB made significant comments on the draft. All authors have read and approved the final manuscript.

## Supplementary Material

Additional file 1 **Table S1**. Distribution of dietary behaviours among the 409 participating first-time mothers and stratified by age, education and household income Click here for file

Additional file 2 **Table S2**. Socio-demographic characteristics associated with poor dietary behaviours in univariate and multivariate analysis Click here for file
