# Supplementary material for: Dietary behaviours during pregnancy: findings from first-time mothers in southwest Sydney, Australia
Source: Int J Behav Nutr Phys Act. 2010 Feb 3;7:13. doi: 10.1186/1479-5868-7-13 (PMC2830165; doi:10.1186/1479-5868-7-13)
Supplement: Additional file 1 — Table S1. Distribution of dietary behaviours among the 409 participating first-time mothers and stratified by age, education and household income [file 1479-5868-7-13-S1.DOC]

Table S1: Distribution of dietary behaviours among the 409 participating first-time mothers and stratified by age, education and household income

| Daily otherwise specified | **Mean**  **(SD), Median** | **n (%)** | **Age** | | | **Education** | | | **Household income** | | |
| --- | --- | --- | --- | --- | --- | --- | --- | --- | --- | --- | --- |
|  |  |  | **< 25** | **≥ 25** | **P^#^** | **Under University** | **University / higher** | **P^#^** | **< $40,000** | **≥ $40,000** | **P^#^** |
|  |  |  | **n (%)** | **n (%)** |  | **n (%)** | **n (%)** |  | **n (%)** | **n (%)** |  |
| **Vegetables (serves)** | 2.3 (1.34), 2 |  |  |  | 0.21 |  |  | 0.23 |  |  | 0.001 |
| Don’t eat or  less than 2 |  | 134 (33) | 67 (38) | 67 (29) |  | 108 (34) | 24 (26) |  | 55 (46) | 79 (27) |  |
| ≥ 2 and < 3 |  | 141 (34) | 55 (31) | 86 (37) |  | 109 (35) | 32 (35) |  | 38 (31) | 103 (36) |  |
| ≥ 3 and < 5 |  | 107 (26) | 41 (24) | 66 (28) |  | 75 (24) | 32 (35) |  | 22 (18) | 85 (30) |  |
| ≥ 5 |  | 27 (7) | 12 (7) | 15 (6) |  | 23 (7) | 4 (4) |  | 6 (5) | 21 (7) |  |
| **Fruit (serves)** | 2.1 (1.38), 2 |  |  |  | 0.002 |  |  | 0.02 |  |  | 0.05 |
| Don’t eat or  less than 1 |  | 46 (11) | 34 (19) | 12 (5) |  | 42 (13) | 3 (3) |  | 21 (17) | 25 (9) |  |
| ≥ 1 and < 2 |  | 109 (27) | 43 (25) | 66 (28) |  | 87 (28) | 22 (24) |  | 26 (21) | 83 (39) |  |
| ≥ 2 and < 4 |  | 201 (49) | 76 (43) | 125 (54) |  | 145 (46) | 55 (60) |  | 60 (50) | 141 (49) |  |
| ≥ 4 |  | 53 (13) | 22 (13) | 31 (13) |  | 41 (13) | 12 (13) |  | 14 (12) | 39 (13) |  |
| **Water (cup)** | 6.1 (3.42), 6 |  |  |  | 0.01 |  |  | 0.02 |  |  | 0.14 |
| 0 to 2 |  | 56 (14) | 28 (17) | 28 (12) |  | 50 (16) | 6 (6) |  | 21 (18) | 35 (13) |  |
| > 2 to 4 |  | 80 (20) | 40 (24) | 40 (18) |  | 61 (20) | 18 (20) |  | 24 (20) | 56 (20) |  |
| > 4 and < 8 |  | 119 (30) | 53 (31) | 66 (29) |  | 91 (30) | 27 (30) |  | 35 (30) | 84 (30) |  |
| ≥8 |  | 142 (36) | 48 (28) | 94 (41) |  | 102 (34) | 40 (44) |  | 37 (32) | 105 (37) |  |
| **Fruit juice (cup)** | 1.5 (1.61), 1 |  |  |  | 0.03 |  |  | 0.14 |  |  | 0.05 |
| Don’t drink |  | 42 (11) | 16 (10) | 26 (12) |  | 32 (11) | 10 (11) |  | 14 (12) | 28 (11) |  |
| ≤ 1 |  | 197 (51) | 75 (47) | 122 (54) |  | 145 (49) | 51 (58) |  | 49 (42) | 148 (55) |  |
| > 1 to 2 |  | 78 (21) | 31 (20) | 47 (21) |  | 60 (21) | 18 (20) |  | 25 (22) | 53 (20) |  |
| > 2 |  | 66 (17) | 37 (23) | 29 (13) |  | 56 (19) | 10 (11) |  | 28 (24) | 38 (14) |  |
| **Soft drinks (cup)** | 1.5 (2.07), 1 |  |  |  | <0.001 |  |  | <0.001 |  |  | 0.27 |
| Don’t drink |  | 74 (19) | 20 (12) | 54 (25) |  | 44 (15) | 30 (36) |  | 21 (18) | 53 (20) |  |
| ≤ 1 |  | 165 (43) | 68 (40) | 97 (46) |  | 121 (41) | 43 (51) |  | 46 (39) | 119 (45) |  |
| > 1 to 2 |  | 64 (17) | 37 (22) | 27 (13) |  | 58 (20) | 5 (6) |  | 24 (21) | 40 (15) |  |
| > 2 |  | 78 (21) | 44 (26) | 34 (16) |  | 72 (24) | 6 (7) |  | 26 (22) | 52 (20) |  |
| **Diet soft drinks (cup)** | 0.5 (1.24), 0 |  |  |  | 0.52 |  |  | 0.07 |  |  | 0.05 |
| Don’t drink |  | 215 (69) | 90 (68) | 125 (69) |  | 156 (67) | 57 (70) |  | 69 (76) | 146 (66) |  |
| ≤ 1 |  | 70 (22) | 28 (21) | 42 (23) |  | 48 (21) | 22 (27) |  | 18 (20) | 52 (23) |  |
| > 1 to 2 |  | 13 (4) | 5 (4) | 8 (4) |  | 11 (5) | 2 (3) |  | 1 (1) | 12 (5) |  |
| > 2 |  | 16 (5) | 9 (7) | 7 (4) |  | 16 (7) | 0 (0) |  | 3 (3) | 13 (6) |  |
| **Sugary drinks* (cup)** | 2.9 (2.67), 2.1 |  |  |  | <0.001 |  |  | <0.001 |  |  | 0.05 |
| Don’t drink |  | 18 (5) | 6 (3) | 12 (5) |  | 14 (4) | 4 (4) |  | 7 (6) | 11 (4) |  |
| Less than 1 |  | 45 (11) | 15 (9) | 30 (13) |  | 28 (9) | 17 (19) |  | 13 (11) | 32 (11) |  |
| ≥ 1 and ≤ 2 |  | 128 (32) | 40 (23) | 88 (39) |  | 85 (28) | 42 (46) |  | 27 (22) | 101 (36) |  |
| > 2 |  | 210 (52) | 112 (65) | 98 (43) |  | 181 (59) | 28 (31) |  | 73 (61) | 137 (49) |  |
| **Cereal per week (times)^** | 4.4 (3.44) 4 |  |  |  | 0.82 |  |  | 0.03 |  |  | 0.35 |
| ≤ 2 |  | 136 (33) | 62 (36) | 74 (32) |  | 96 (30) | 39 (42) |  | 44 (36) | 92 (32) |  |
| 2-6 |  | 88 (22) | 32 (18) | 56 (24) |  | 68 (22) | 19 (21) |  | 26 (22) | 62 (22) |  |
| >6 |  | 185 (45) | 81 (46) | 104 (44) |  | 151 (48) | 34 (37) |  | 51 (42) | 134 (46) |  |
| **Pasta per week (times) ^&^** | 4.5 (4.4) 3 |  |  |  | 0.03 |  |  | <0.001 |  |  | 0.27 |
| ≤ 2 |  | 151 (37) | 73 (42) | 78 (33) |  | 127 (40) | 23 (25) |  | 50 (41) | 101 (35) |  |
| 2-6 |  | 151 (37) | 65 (37) | 86 (37) |  | 121 (39) | 29 (32) |  | 42 (35) | 109 (38) |  |
| >6 |  | 107 (26) | 37 (21) | 70 (30) |  | 67 (21) | 40 (43) |  | 29 (24) | 78 (27) |  |
| **Milk per day (cup) ^$^** | 2.1 (1.51) 2 |  |  |  | 0.26 |  |  | 0.04 |  |  | 0.66 |
| ≤ 1 |  | 157 (39) | 65 (37) | 92 (39) |  | 120 (38) | 36 (39) |  | 48 (40) | 109 (38) |  |
| 1-2 |  | 124 (30) | 48 (28) | 76 (33) |  | 88 (28) | 36 (39) |  | 37 (30) | 87 (30) |  |
| >2 |  | 128 (31) | 62 (35) | 66 (28) |  | 107 (34) | 20 (22) |  | 36 (30) | 92 (32) |  |
| **Processed meat per week**  **(times)** | 2 (3.99) 1 |  |  |  | 0.14 |  |  | 0.66 |  |  | 0.91 |
| Don’t eat meat |  | 115 (28) | 47 (27) | 68 (29) |  | 90 (29) | 24 (26) |  | 39 (32) | 76 (26) |  |
| ≤ 1 |  | 139 (34) | 52 (30) | 87 (37) |  | 101 (32) | 38 (41) |  | 32 (27) | 107 (37) |  |
| >1 |  | 155 (38) | 76 (43) | 79 (34) |  | 124 (39) | 30 (33) |  | 50 (41) | 105 (37) |  |
| **Fast food/ take away per week (times)** | 1.2 (1.31) 1 |  |  |  | 0.003 |  |  | 0.006 |  |  | 0.01 |
| <1 |  | 151 (37) | 53 (31) | 98 (42) |  | 105 (34) | 46 (50) |  | 43 (36) | 108 (38) |  |
| 1-2 |  | 207 (51) | 92 (53) | 115 (50) |  | 167 (54) | 39 (42) |  | 54 (45) | 153 (53) |  |
| >2 |  | 47 (12) | 28 (16) | 19 (8) |  | 39 (12) | 7 (8) |  | 22 (19) | 25 (9) |  |
| **Chips per week (times)** | 2.4 (4.17) 1 |  |  |  | 0.01 |  |  | <0.001 |  |  | 0.35 |
| <1 |  | 125 (31) | 50 (29) | 75 (32) |  | 87 (28) | 38 (41) |  | 35 (29) | 90 (31) |  |
| 1-2 |  | 166 (41) | 59 (34) | 107 (46) |  | 124 (40) | 41 (45) |  | 47 (39) | 119 (42) |  |
| >2 |  | 116 (28) | 65 (37) | 51 (22) |  | 102 (32) | 13 (14) |  | 39 (32) | 77 (27) |  |

*Sugary drinks = soft drink + fruit juice; ^breakfast cereal including ready-made, home-made or cooked cereal. **^&^**including pasta, rice or noodles;

^$^ including mild used in tea, or coffee, cow milk, soy milk, milk on cereal, flavoured milks

**^#^**Mantel-Haenszel chi-squared tests for trend in proportions
