# Supplementary material for: Dietary behaviours during pregnancy: findings from first-time mothers in southwest Sydney, Australia
Source: Int J Behav Nutr Phys Act. 2010 Feb 3;7:13. doi: 10.1186/1479-5868-7-13 (PMC2830165; doi:10.1186/1479-5868-7-13)
Supplement: Additional file 2 — Table S2. Socio-demographic characteristics associated with poor dietary behaviours in univariate and multivariate analysis [file 1479-5868-7-13-S2.DOC]

**Table S2: Socio-demographic characteristics associated with poor dietary behaviours in univariate and multivariate analysis**

| **Variables** | **Vegetables per day** | | | | **Fruit per day** | | | | **Soft drink per day** | | | | **Fast food/takeaway per week** | | | |
| --- | --- | --- | --- | --- | --- | --- | --- | --- | --- | --- | --- | --- | --- | --- | --- | --- |
|  | < 2 serves  N=134  n (row%) | P^1^ | ARR.*  95%CI | P^#^ | <2 serves  N=155  n (row%) | P^1^ | ARR. 95%CI | P^#^ | >1 cup  N=142  n (row%) | P^1^ | ARR#. 95%CI | p^#^ | >2 times  N=47  n (row%) | P^1^ | ARR. 95%CI | P^#^ |
| **Age** |  | 0.07 |  |  |  | 0.06 |  |  |  | <0.001 |  | 0.12 ^e^ |  | 0.02 |  | 0.03 ^h^ |
| ≤ 24 | 67 (38) |  |  |  | 77 (44) |  |  |  | 81 (48) |  | 1 |  | 28 (16) |  | 1 |  |
| 25-29 | 36 (26) |  |  |  | 46 (33) |  |  |  | 42 (33) |  | 0.9 (0.7-1.3) |  | 14 (10) |  | 0.7 (0.4-1.4) |  |
| ≥ 30 | 31 (33) |  |  |  | 32 (34) |  |  |  | 19 (23) |  | 0.7 (0.5-1.1) |  | 5 (5) |  | 0.4 (0.1-0.9) |  |
| **Marital status** |  | 0.14 |  |  |  | 0.99 |  |  |  | 0.02 |  | 0.95 ^e^ |  | 0.006 |  | 0.04 ^i^ |
| Married /de-facto  partner | 111 (31) |  |  |  | 134 (38) |  |  |  | 113 (35) |  | 1 |  | 35 (10) |  | 1 |  |
| Never married | 22 (42) |  |  |  | 20 (38) |  |  |  | 27 (52) |  | 1.1 (0.8-1.5) |  | 12 (23) |  | 1.9 (1.1-3.7) |  |
| **Employment status** |  | 0.006 |  | 0.25 ^a^ |  | 0.18 |  |  |  | 0.008 |  | 0.16 ^e^ |  | 0.37 |  |  |
| Employed | 58 (26) |  | 1 |  | 83 (37) |  |  |  | 62 (30) |  | 1 |  | 21 (10) |  |  |  |
| Unemployed | 36 (41) |  | 1.2 (0.8-1.8) |  | 28 (32) |  |  |  | 40 (47) |  | 1.3 (0.9-1.9) |  | 11 (13) |  |  |  |
| Home duties/  Student/other | 39 (41) |  | 1.2 (0.9-1.8) |  | 43 (45) |  |  |  | 40 (45) |  | 1.3 (0.9-1.8) |  | 14 (15) |  |  |  |
| **Education** |  | 0.08 |  |  |  | 0.02 |  | 0.03 ^c^ |  | <0.001 |  | 0.001^f^ |  | 0.04 |  | 0.81 ^j^ |
| Completed  primary school to  school certificate | 33 (38) |  |  |  | 41 (48) |  | 1 |  | 47 (56) |  | 1 |  | 15 (17) |  | 1 |  |
| HSC to TAFE  certificate or  diploma | 75 (33) |  |  |  | 88 (38) |  | 0.9 (0.6-1.2) |  | 83 (39) |  | 0.8 (0.6-1.0) |  | 24 (11) |  | 0.9 (0.5-1.8) |  |
| University | 24 (26) |  |  |  | 25 (27) |  | 0.6 (0.4-0.8) |  | 11 (13) |  | 0.3 (0.2-0.6) |  | 7 (8) |  | 0.9 (0.3-2.3) |  |
| **Country of birth** |  | 0.37 |  |  |  | 0.02 |  | 0.14 ^d^ |  | <0.001 |  | 0.03 ^g^ |  | 0.96 |  |  |
| Australia | 86 (31) |  |  |  | 40 (30) |  | 1 |  | 30 (24) |  | 1 |  | 16 (12) |  |  |  |
| Other | 48 (36) |  |  |  | 115 (42) |  | 1.3 (0.9-1.7) |  | 112 (44) |  | 1.8 (1.1-3.1) |  | 31 (11) |  |  |  |
| **Household income** |  | <0.001 |  | 0.003 ^b^ |  | 0.97 |  |  |  | 0.03 |  | 0.65 ^e^ |  | 0.02 |  | 0.24 ^j^ |
| <$40,000 | 55 (46) |  | 1 |  | 47 (39) |  |  |  | 50 (43) |  | 1 |  | 22 (19) |  | 1 |  |
| $40,000-79,999 | 45 (33) |  | 0.8 (0.6-1.1) |  | 52 (38) |  |  |  | 52 (41) |  | 1.3 (1.0-1.7) |  | 10 (7) |  | 0.5 (0.2-0.9) |  |
| ≥ $80,000 | 34 (23) |  | 0.6 (0.4-0.9) |  | 56 (37) |  |  |  | 40 (29) |  | 1.2 (0.8-1.8) |  | 15 (10) |  | 0.7 (0.4-1.4) |  |

P^1^ : Bivariate analysis using chi-squared tests or Mantel-Haenszel chi-squared tests for trend in proportions when appropriate; ARR*=adjusted risk ratio

P^#^ : Multivariate analysis using logistic regression

a. adjusted for household income; b. adjusted for employment; c. adjusted for country of birth; d. adjusted for education;

e. adjusted for education and country of birth; f. adjusted for country of birth; g. adjusted for education; h. adjusted for marital status;

i. adjusted for age; j. adjusted for age and marital status
